# Personality traits and procrastination among medical students: the mediating role of trait emotional intelligence

**DOI:** 10.3389/fpsyg.2026.1725503

**Published:** 2026-01-23

**Authors:** Ayoob Lone, Mazen Raed Mufarreh

**Affiliations:** 1 Department of Clinical Neurosciences, College of Medicine, King Faisal University, Al-Ahsa, Saudi Arabia; 2 College of Medicine, King Faisal University, Al-Ahsa, Saudi Arabia

**Keywords:** academic procrastination, medical students, personality traits, Saudi Arabia, trait emotional intelligence

## Abstract

**Introduction:**

Academic procrastination is a prevalent issue among medical students, often influenced by personality traits and emotional regulation abilities. Trait Emotional intelligence has been suggested as a potential mediator in the relationship between personality and procrastination, but this relationship remains underexplored in Saudi Arabia. This study examined the mediating role of trait emotional intelligence in the association between Big Five personality traits and academic procrastination among medical students.

**Methods:**

A cross-sectional survey was conducted among 317 undergraduate medical students at King Faisal University, Saudi Arabia, using stratified random sampling. Validated instruments were employed, including the Academic Procrastination Scale–Short Form, Big Five Inventory–10, and Brief Emotional Intelligence Scale. Structural Equation Modeling with bootstrapping (5,000 samples) was used to assess direct and indirect effects.

**Results:**

The findings of this study revealed that openness to experience, conscientiousness, and agreeableness were significantly and negatively associated with academic procrastination. In contrast, extraversion and neuroticism exhibited significant positive associations. Trait emotional intelligence demonstrated a strong negative direct effect on academic procrastination. Mediation analyses indicated that trait emotional intelligence partially mediated the relationship between openness and procrastination, and between conscientiousness and procrastination. A full mediating effect was observed between agreeableness and procrastination. No significant mediation effects were found for extraversion or neuroticism.

**Conclusion:**

The findings underscore the pivotal role of personality traits and trait emotional intelligence in shaping procrastination behaviors among medical students. Specifically, trait emotional intelligence functions as a key mediator in reducing the negative impact of certain personality traits on procrastination. These results suggest that targeted interventions aimed at enhancing trait emotional intelligence may mitigate academic procrastination and improve performance outcomes in medical education settings.

## Introduction

Procrastination is a psychological and behavioral phenomenon characterized by the intentional delay of intended actions despite awareness of potential negative consequences (Steel, 2007). It is a widespread behavior observed across multiple life domains and is associated with adverse outcomes, including elevated stress, reduced productivity, and impaired well-being. Contemporary perspectives conceptualize procrastination not merely as poor time management but as a self-regulation failure influenced by motivational, cognitive, and emotional factors (Rozental and Carlbring, 2014; Prihadi et al., 2018).

In recent years, academic procrastination has gained growing attention in research (Ramadhani and Putrie, 2023), Within educational contexts, procrastination manifests as academic procrastination, defined as the deliberate postponement of academic tasks such as studying, completing assignments, or preparing for examinations (Balkis, 2013; Özberk and Kurtca, 2021; Rahimi and Vallerand, 2021). Academic procrastination has emerged as a major concern in modern educational systems due to its negative impact on performance, workload management, and psychological health (Balkis, 2013; Kljajic and Gaudreau, 2018; Martinie and Shankland, 2024; Hayat et al., 2020a; Ramadhani et al., 2026). Although procrastination has traditionally been associated with deficits in self-regulation, research suggests that it is a multifactorial process, influenced by both internal factors (e.g., personality, emotional regulation) and external academic demands (Prihadi et al., 2018; Sirois, 2023).

Medical students face particularly high academic and emotional demands, including intensive coursework, frequent evaluations, and early clinical exposure, which may exacerbate tendencies toward procrastination (Shah et al., 2017). Empirical studies report that approximately 29.25% of medical students experienced academic procrastination (Hayat et al., 2020b). Moreover, Shankar et al. (2017) indicated that frequency of academic procrastination was nearly 33% among undergraduate medical students. A recent study conducted by Sönmez et al. (2023) reported 45% of the sample had a tendency to procrastinate. It seems the percentage of academic procrastination appears to be on the rise among students. These findings highlight the critical need to investigate the psychological factors underlying procrastination in this population.

Previous studies highlighted personality as an important factor linked to academic procrastination (Golmohammadian, 2015; Sandhya and Gopinath, 2019; Ocansey et al., 2022; Kokkinos et al., 2023; Cheng et al., 2023). Personality traits represent stable patterns of thinking, feeling, and behaving that influence how individuals respond to environmental demands, including academic challenges (DeYoung, 2015). These traits are generally stable and influence how a person reacts to various circumstances, shapes their decisions, and interacts with others. Certain personality traits from established personality models can have a significant influence on procrastination behaviors, particularly in academic settings. Among internal predictors of academic procrastination, the Big Five personality model (extraversion, agreeableness, conscientiousness, neuroticism, and openness to experience) has been most widely studied (McCrae and Costa, 1997).

Empirical evidence consistently identifies conscientiousness as a key protective factor against procrastination due to its association with self-discipline, planning, and task persistence (Steel and Klingsieck, 2016; Awad et al., 2023; Zhang, 2024). Neuroticism is a reliable predictor of increased procrastination, reflecting vulnerability to anxiety, emotional instability, and avoidance behaviors (Chen et al., 2019). Findings for other traits are less consistent: extraversion may increase procrastination in some contexts, whereas agreeableness and openness to experience generally show negative or mixed associations with procrastination (Aremu et al., 2011; Boysan and Kiral, 2017). Overall, conscientiousness and neuroticism emerge as the most critical traits for understanding procrastination in academic settings.

While personality traits partially explain academic procrastination, they do not fully capture the emotional processes that influence students’ task engagement and avoidance behaviors. Emotional intelligence (EI) which reflects the ability to perceive, understand, regulate, and utilize emotions (Salovey and Mayer, 1990; Petrides, 2001), is introduced as a complementary construct to address this gap. By examining EI alongside the Big Five traits, the present study investigates whether emotional competencies mediate the relationship between stable personality traits and procrastination, providing a more comprehensive understanding of why some students with “risk” personality profiles may still avoid procrastination, while others do not. This rationale underscores the relevance of EI as an additional psychological factor when personality traits alone are insufficient to explain procrastination behaviors in medical students.

Previously, EI was framed as an ability-based construct comparable to general intelligence (Salovey and Mayer, 1990). It was conceptualized as an individual’s mental capacity and emotional knowledge required to perceive, recognize, and comprehend emotions accurately (Mayer et al., 1999). Accordingly, the ability model of EI was aligned with cognitive intelligence and regarded as universally adaptive (Petrides et al., 2010). Later, two conceptually distinct models of EI emerged: Trait emotional intelligence and the mixed model (Fiori and Vesely-Maillefer, 2018; O’Connor et al., 2019). Trait EI is defined as “a constellation of emotional self-perceptions located at the lower levels of personality hierarchies (Petrides et al., 2007), whereas the mixed model conceptualizes EI as an integration of personal traits, social skills, and competencies (O’Connor et al., 2019). In this study, we adopt a trait/self-report perspective of EI, consistent with the instruments employed (BEIS-10), to assess students’ self-perceived emotional competencies in recognizing, regulating, and utilizing emotions.

Prior studies examining the relationship between EI and academic procrastination have reported mixed findings. Some studies have demonstrated a significant negative association between higher EI and procrastination, predominantly conceptualizing EI as a trait and assessing it using self-report measures (Deniz et al., 2009; Guo et al., 2019). In contrast, other studies have reported indirect or context-dependent effects of EI on procrastination (Hen and Goroshit, 2014; Kamran and Fatima, 2013), often employing different theoretical frameworks or operationalization’s of EI. These variations in whether EI is conceptualized as an ability or a trait may partly explain the inconsistencies observed in the literature. Accordingly, by treating EI as a self-reported trait, the present study aims to clarify how emotional competencies mediate or moderate the influence of personality traits on academic procrastination among medical students.

### The present study

Despite extensive research demonstrating that personality traits are associated with academic procrastination, evidence suggests that these traits alone do not fully explain procrastination behavior, particularly in demanding academic contexts such as medical education. Medical students face intense academic workloads, clinical responsibilities, and emotional stressors, making them especially vulnerable to procrastination-related difficulties. However, research examining these relationships among medical students in Saudi Arabia remains limited, underscoring the need for context-specific investigation.

Drawing on the Big Five personality framework, prior studies consistently identify conscientiousness and neuroticism as the strongest predictors of academic procrastination, with conscientiousness showing a robust negative association and neuroticism demonstrating a positive association (Steel, 2007; Kim et al., 2017; Chen et al., 2019). Evidence regarding extraversion, agreeableness, and openness to experience has been more mixed, suggesting that additional psychological mechanisms may influence how these traits relate to procrastination. Accordingly, the present study first examines the direct effects of personality traits on academic procrastination (H1–H5).

Beyond direct effects, personality traits are also known to shape individuals’ emotional competencies, including emotion perception, regulation, and utilization. Trait emotional intelligence, conceptualized as a set of self-perceived emotional abilities embedded within personality hierarchies (Petrides et al., 2007), provides a theoretically grounded construct for capturing these emotional self-regulatory capacities. Empirical evidence indicates that conscientiousness, agreeableness, and openness are positively associated with EI (Vernon et al., 2008); whereas neuroticism shows a strong negative association (Hjalmarsson and Dåderman, 2022). Therefore, the present study hypothesizes associations between each personality trait and EI (H6–H10).

Trait EI has been linked to more effective emotion regulation, stress management, and self-control (Trigueros et al., 2020), all of which are critical for reducing avoidance behaviors such as procrastination. Prior studies suggest that higher EI is associated with lower academic procrastination, although findings vary depending on how EI is operationalized (Deniz et al., 2009; Guo et al., 2019; Hen and Goroshit, 2014). Consistent with a trait-based framework, the present study hypothesizes a negative association between EI and academic procrastination (H11).

**Figure 1:**
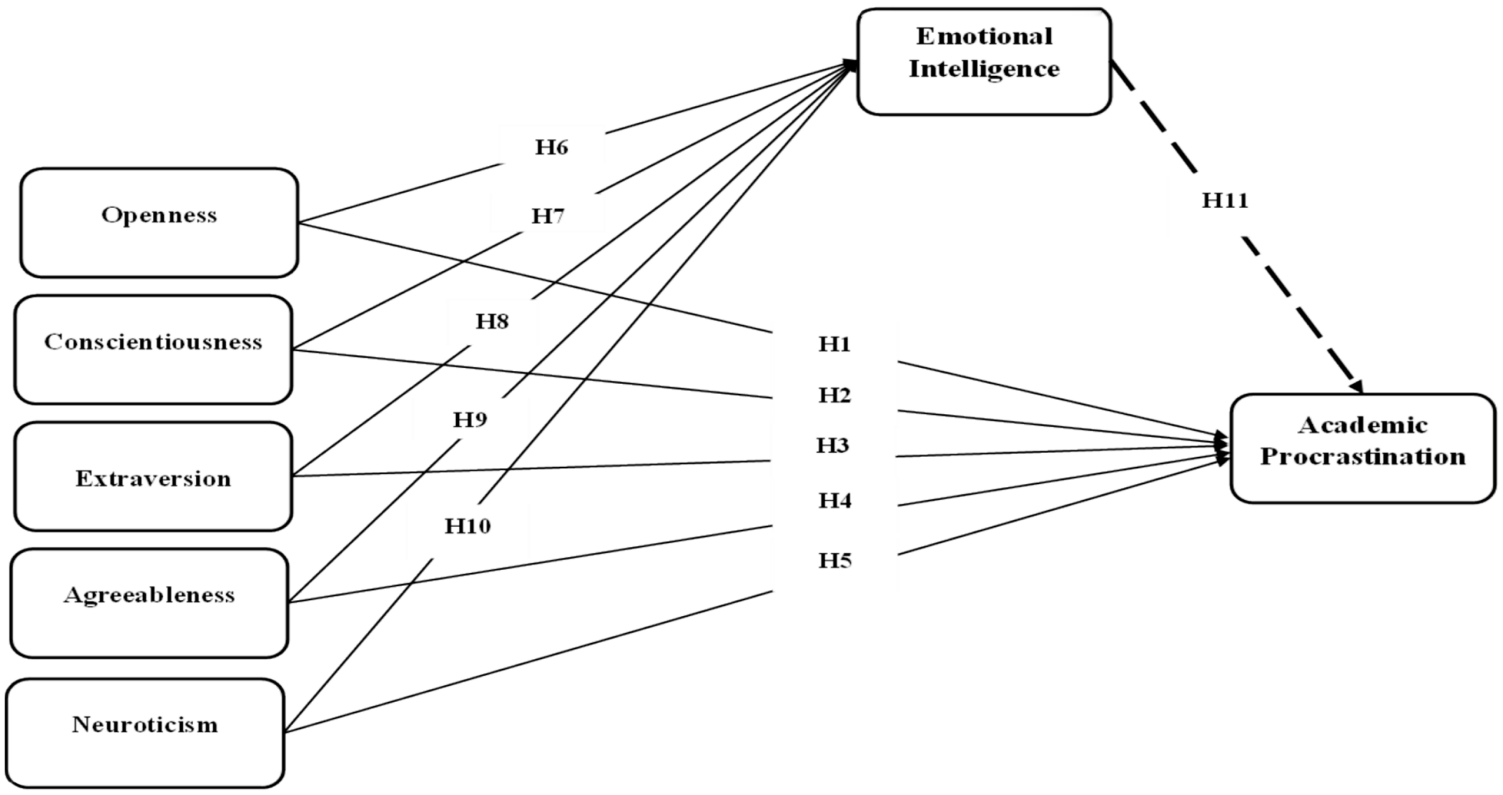
Conceptual model.

Taken together, this study proposes a mediation model in which trait emotional intelligence serves as an explanatory mechanism linking personality traits to academic procrastination. Specifically, trait emotional intelligence is expected to attenuate the maladaptive influence of neuroticism while enhancing the protective effects of conscientiousness, agreeableness, and openness. This mediation approach aligns with established methodological recommendations for uncovering underlying psychological processes (Fairchild and MacKinnon, 2009) and is illustrated in Figure 1.

## Materials and methods

### Study design

The present study employed a cross-sectional design to gather data from Saudi medical undergraduate’s students from April to August 2025. The study received ethical approval from the Deanship of Scientific Research at King Faisal University in Al-Ahsa, Saudi Arabia (KFU-REC-2025-JAN-ETHICS3015), and this study is in accordance with the principles outlined in the Declaration of Helsinki for research involving human participants. All participants were provided with detailed information about the study’s purposes and goals, and the survey was carried out only after fulfilling all necessary ethical obligations.

### Participants and sampling

The study sample consisted of undergraduate medical students from the College of Medicine, King Faisal University, Al-Ahsa, Saudi Arabia. A stratified approach was used to ensure internal balance across academic years. Students were grouped according to their year of study, and invitations to participate were distributed to eligible students within each group. Participation was voluntary, and no probability-based random sampling was applied. The stratification was implemented solely to facilitate internal comparability across different academic years and was not intended to achieve population-level representativeness or support generalization beyond the study sample.

### Sample size calculation

A sample of 300 participants was deemed appropriate for conducting Structural Equation Modelling (Memon et al., 2020). According to methodological guidelines, SEM requires relatively large sample sizes to ensure stable and reliable parameter estimation. Previous literature suggested that the adequacy of a sample size can be determined based on the total number of observed variables (Rokhman et al., 2022). Memon et al., 2020 recommended that the minimum number of participants should be at least five times the number of variables, preferably ten times (Yew et al., 2022) and ideally 15 to 20 times, as suggested by Hair et al. (2014a). In this study, eleven variables were examined. With a total of 317 respondents, the sample size represents 29.0 times the number of the variables, thereby exceeding the commonly cited recommendations. Based on the total number of 1,639 students enrolled in College of Medicine, King Faisal University, a single University in Eastern Governorate of Saudi Arabia, the sample size was determined by using Yamane’s formula (Adam, 2020). The applied formula calculated a total sample size of 317 participants, based on an expected margin of error of 0.05, which was deemed appropriate for the present study.

### Measures

In order to achieve the study’s objectives, a variety of measures were utilized. The Academic Procrastination Scale-Short Form (APS-SF) was employed to assess procrastination tendencies, while the Big Five Personality factors were used to evaluate personality traits. Trait emotional intelligence was measured using the BEIS-10. Furthermore, a demographic questionnaire created developed by the researchers was also included.

#### Academic procrastination scale-short form (APS-SF)

Academic Procrastination Scale- Short Form was used to measure the severity of academic procrastination (Yockey, 2016) among medical students. This scale consists of 5 items rated on a 5-point Likert scale ranging from 1 = disagree to 5 = agree. Score ranges from 5 to 25, with higher score indicating an increased level of academic procrastination. Brando-Garrido et al. (2020) performed a psychometric evaluation of the APS-SF with a sample of nursing students in Spain, finding an internal consistency of *α* = 0.87 and demonstrating satisfactory convergent validity with several other procrastination measures. In this study, the internal consistency reliability for this measure, as indicated by Cronbach’s alpha, was 0.89 for the current sample.

#### Big five personality factors

Personality was evaluated using the Big Five Inventory-10 (BFI-10) (Rammstedt and John, 2007). This inventory measures five distinct dimensions of personality: Extraversion (items 1 and 6), Agreeableness (items 2 and 7), Conscientiousness (items 3 and 8), Neuroticism (items 4 and 9), and Openness to Experience (items 5 and 10). Each dimension consists of two items, one positively keyed and the other negatively keyed. Participants respond on a 5-point Likert scale, where 1 represents “strongly disagree” and 5 represents “strongly agree.” The total score for each participant is calculated by summing the individual subscale scores, which range from 10 to 50. Higher scores indicate stronger traits in the respective personality dimension, with a greater score reflecting a more pronounced level of that personality characteristic. In the present study, the internal consistency reliability (Cronbach’s alpha) for the different subscales of the Big Five Inventory-10 was reported as follows: Extraversion (0.92), Agreeableness (0.81), Conscientiousness (0.84), Neuroticism (0.82), and Openness (0.81). These values indicate varying degrees of reliability across the subscales, with Extraversion showing relatively higher internal consistency, while conscientiousness, agreeableness, neuroticism and Openness exhibited a lower level of reliability. These findings suggest that while some personality dimensions are measured more consistently, others may require further attention or refinement for better reliability.

#### Brief emotional intelligence scale (BEIS-10)

Trait emotional intelligence was assessed using the BEIS-10 (Davies et al., 2010), a 10-item instrument based on the Schutte Emotional Intelligence Scale and the emotional intelligence model proposed by Salovey and Mayer (1990). The BEIS-10 measures five distinct components of emotional intelligence: the utilization of emotions, awareness of one’s own emotions, regulation of one’s own emotions, recognition of others’ emotions, and regulation of others’ emotions (Davies et al., 2010). Respondents rated each item on a 5-point Likert scale ranging from 1 (strongly agree) to 5 (strongly disagree), with higher scores indicating a lower level of emotional intelligence. The total emotional intelligence score was derived by reversing the scores to ensure that higher emotional intelligence scores correspond to a lower emotional intelligence. The BEIS-10 demonstrated strong internal consistency, with Cronbach’s alpha values ranging from 0.85 to 0.89, reflecting its excellent reliability for measuring trait emotional intelligence in the study’s sample (Davies et al., 2010).

#### Demographic questionnaire

The demographic questionnaire was also utilized in this study collected a range of information regarding participants’ personal and family backgrounds. This included basic demographic details such as age, gender, as well as educational qualification. In addition, the questionnaire gathered information about participants’ family structure and living conditions, including their place of residence, family type, income level, and housing status. This comprehensive data allowed for a more detailed understanding of the participants’ socio-economic and personal context.

### Procedure

The data was gathered using structured self-administered questionnaires. The questionnaires were distributed by trained medical students who underwent specific training prior to the data collection process. The data collectors were supervised by their respective supervisors throughout the entire process to ensure consistency and accuracy. The questionnaires were distributed to all selected study participants with the assistance of the data collectors, who provided guidance and support as needed. The distribution took place in the college campus, and prior to completing the questionnaires, the participants were informed about the purpose and goals of the study.

### Statistical analysis

After verifying the quality and consistency of the data, it was coded and entered into EPI data version 3.1. Subsequently, the dataset was exported to SPSS version 26 for analysis. Initially, frequency distributions and descriptive statistics, including means and standard deviations, were calculated. Pearson’s correlation coefficients were calculated to assess the bivariate relationships among the key variables: the Big Five personality traits, trait emotional intelligence and academic procrastination. Subsequently, Partial least squares-structural equation modeling (PLS-SEM) was employed using bootstrapping with 5,000 resamples to evaluate the hypothesized structural relationships and the mediating effect of trait emotional intelligence. This approach enabled simultaneous testing of direct and indirect effects. Model fit was assessed using the standardized root mean square residual (SRMR), with a value of 0.065, indicating an acceptable fit (< 0.08). Multicollinearity was examined using variance inflation factor (VIF), with all values ranging between 1.016 and 3.096, below the critical threshold of 5. Measurement model validity was confirmed through factor loadings (>0.70), composite reliability (CR) values ranging from 0.716 to 0.919, average variance extracted (AVE) between 0.558 and 0.891, establishing convergent validity. Discriminant validity was evaluated using: Fornell and Larcker’s criterion, where the square root of AVE exceeded inter-construct correlations, Heterotrait-Monotrait ratio (HTMT), with all values below 0.90. For mediation analysis, indirect effects were tested using bootstrapped confidence intervals, following the guidelines by Zhao et al. (2010), and Hays and Scharkow (2013). A mediation effect was considered significant when the bias-corrected 95% confidence interval did not include zero.

## Results

Table 1 summarizes the sociodemographic characteristics of the study sample, which comprised 317 undergraduate medical students from the College of Medicine, King Faisal University, Al-Ahsa, Saudi Arabia. The sample included 149 males (47.0%) and 168 females (53.0%), with a mean age of 21.42 years. Most participants were enrolled in the earlier academic years, particularly the second year (27.8%). The majority resided in urban areas (74.4%), and nearly half reported a monthly family income exceeding 15,001 SAR (47.9%). These variables are presented descriptively to characterize the sample and were not included as predictors or covariates in the primary analyses.

**Table 1:** Demographic characteristics of participants.

Characteristics	*N* (317)	*%*
Gender		
Male	149	47.0
Female	168	53.0
Age (Mean = 21.42; SD = 1.60)		
Academic year		
1st	60	18.9
2nd	88	27.8
3rd	67	21.1
4th	53	16.7
5th	49	15.5
Family status		
Nuclear	173	54.6
Joint	144	45.4
Area of residence		
Urban	236	74.4
Rural	81	25.6
Family income		
<5000SAR	34	10.7
5,001–10,000	80	25.2
10,001–15,000	51	16.1
>15,001	152	47.9
Type of stay		
Living with family	236	74.4
University housing	60	18.9
Sharing apartment	17	5.4
Personal apartment	4	1.3
GPA in previous semester		
4.5–5	113	35.6
4–4.4	103	32.5
3–3.9	81	25.6
2–2.9	17	5.4
< 2	3	0.9

### Measurement model

**Figure 2:**
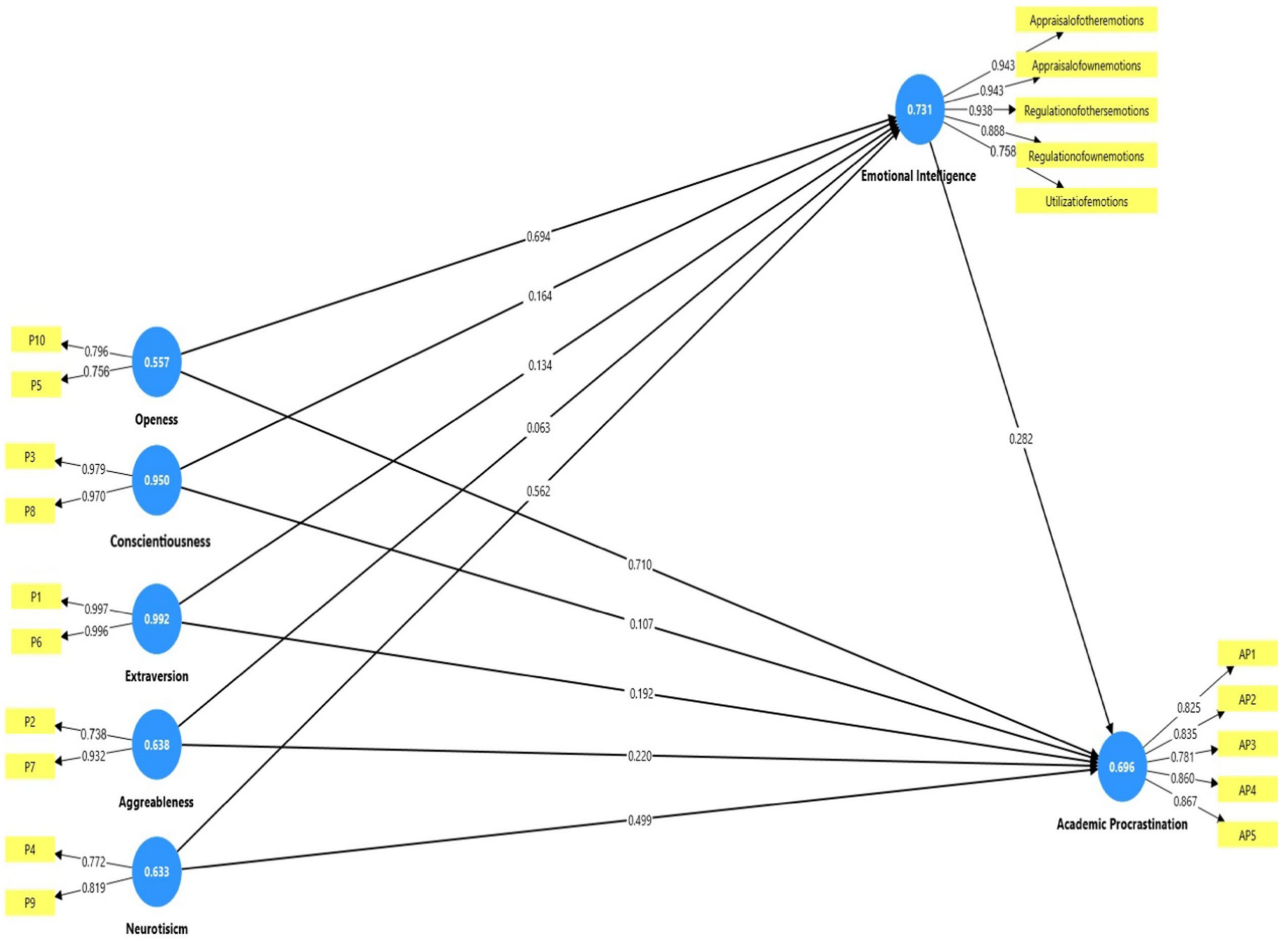
Measurement model.

To ensure the psychometric properties of the measurement model (Figure 2), the authors initially checked the reliability and validity. For this purpose, the assessment of composite reliability (CR), convergent, and discriminant validity was reported in Table 2. The results revealed that the factor loadings were above the threshold value of 0.70 (Ali et al., 2018). The Composite reliability ranges from 0.716 to 0.919, satisfying the accepted threshold value of 0.70 (Hair et al., 2014b). Table 2 further shows that the value of average variance extracted (AVE) ranged from 0.558 to 0.891, providing sufficient evidence for convergent validity (Hair et al., 2010). Further, the discriminant validity was assessed using Fornell and Larcker’s (1981) criteria. Who argued that the square root of AVE presented on the diagonal in Table 3, should be greater than the related inter-construct correlations. The authors also used the Heterotrait-monotrait ratio, which is considered an advanced method in detecting validity issues. Table 4 indicates that all the HTMT values are below the recommended threshold value of 0.90, indicating a significant link between indicators and constructs (Henseler et al., 2015).

**Table 2:** Cronbach’s alpha, composite reliability and average variance extracted.

Construct	Cronbach’s alpha	Composite reliability	Average variance extracted
Academic procrastination	0.892	0.919	0.696
Trait emotional intelligence	0.937	0.910	0.850
Openness	0.807	0.716	0.558
Conscientiousness	0.847	0.914	0.853
Extraversion	0.922	0.916	0.891
Agreeableness	0.811	0.772	0.637
Neuroticism	0.822	0.776	0.634

**Table 3:** Discriminant validity (Fornell and Lacker).

	Construct	1	2	3	4	5	6	7
1	Academic procrastination	0.834						
2	Trait emotional intelligence	−0.261	0.975					
3	Agreeableness	−0.221	0.064	0.798				
4	Conscientiousness	−0.107	0.152	0.007	0.975			
5	Extraversion	0.192	−0.112	−0.003	−0.013	0.996		
6	Neuroticism	0.478	−0.587	−0.093	−0.063	−0.144	0.796	
7	Openness	−0.689	0.721	0.189	0.116	−0.173	−0.738	0.747

**Table 4:** Heterotrait-Monotrait (HTMT) ratio for discriminant validity.

	Construct	1	2	3	4	5	6	7
1	Academic procrastination							
2	Trait emotional intelligence	0.273						
3	Agreeableness	0.294	0.124					
4	Conscientiousness	0.124	0.154	0.059				
5	Extraversion	0.200	0.112	0.043	0.022			
6	Neuroticism	0.746	0.805	0.182	0.135	0.223		
7	Openness	0.493	0.632	0.585	0.267	0.373	0.499	

### Structural model

Researchers applied bootstrapping with 5,000 iteration in order to test the structural model and proposed hypotheses by evaluating significance of path coefficients. The standardized root mean square residual (SRMR) was used as model fit criterion as proposed by Henseler et al. (2015). According to the Henseler et al. (2015), a value of *<*0.08 of SRMR highlights an acceptable model fit, whereas 0 value of SRMR indicates a perfect fit. The results revealed that the value SRMR for the present study is 0.065, indicating adequate fit for the data. The multicollinearity of the model was tested with the range of VIFs, which falls between 1.016 to 3.096, and are found below the threshold value of 5 (Hair et al., 2012). To evaluate the structural model, authors used coefficient of determination (R^2^), path coefficient (*β*), and significance level (Hair et al., 2014a). To evaluate the significance of path coefficients and standard error of the model, a t-statistics was generated with bootstrapping procedure engaging 5,000 resamples. The *p-*values attained allows the testing of acceptance and rejection of formulated hypotheses. The metrics of *R*^2^ reveals the relation between exogenous and endogenous constructs and test the ability of the model for accuracy of predictions. The values of *R*^2^ below 0.25 indicate a suggest inadequate accuracy, below 0.50 indicate moderate accuracy, and below 0.75 suggest a significant level of predictive accuracy (Merli et al., 2019). The value of *R*^2^ for academic procrastination is 0.509 and for trait emotional intelligence is 0.503 signifying significant predictive accuracy.

**Figure 3:**
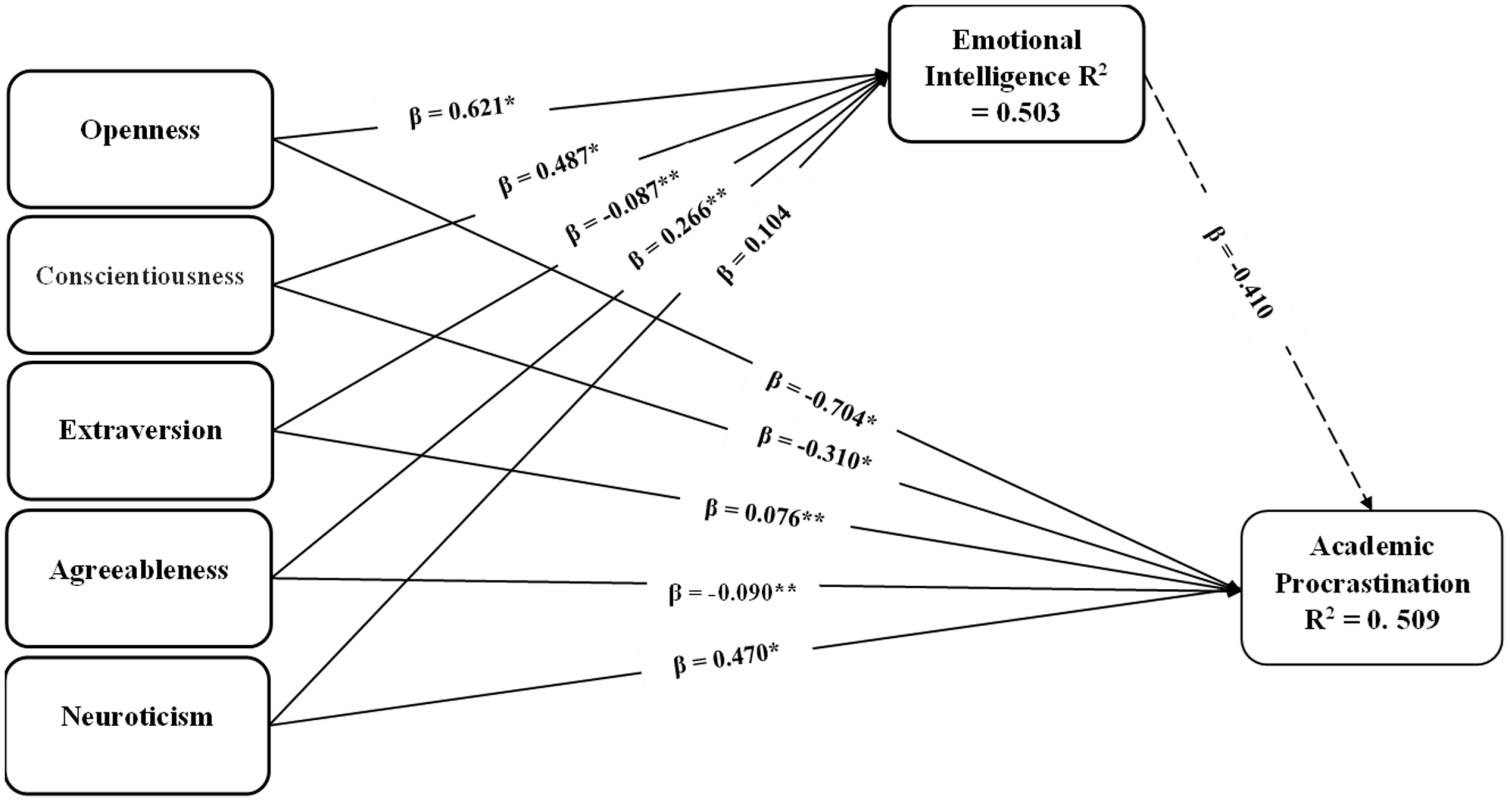
Structural model.

The bootstrapping analysis summarized in Table 5 and illustrated in Figure 3 indicates that openness, conscientiousness, and agreeableness exert significant negative effects on academic procrastination, whereas extraversion and neuroticism are associated with higher levels of procrastination. Trait emotional intelligence also shows a significant negative association with academic procrastination. With respect to predictors of trait emotional intelligence, openness, conscientiousness, and agreeableness demonstrated significant positive effects, while extraversion showed a small negative effect and neuroticism was not a significant predictor. Together, these findings support the proposed mediation model, suggesting that trait emotional intelligence partially transmits the effects of certain personality traits on academic procrastination.

**Table 5:** Hypothesis testing.

Relationships	*β*	*t*-statistics	*p*-value	Result
TEI ➔ AP	−0.410	5.303	0.000	Supported -ve
Openness ➔ AP	−0.704	8.025	0.000	Supported −ve
Openness ➔ TEI	0.621	7.623	0.000	Supported +ve
Conscientiousness ➔ AP	−0.310	3.435	0.002	Supported −ve
Conscientiousness ➔ TEI	0.487	5.897	0.000	Supported +ve
Extraversion ➔ AP	0.076	1.982	0.024	Supported +ve
Extraversion ➔ TEI	−0.087	1.899	0.028	Supported −ve
Agreeableness ➔ AP	−0.090	1.987	0.043	Supported −ve
Agreeableness ➔ TEI	0.266	2.196	0.016	Supported +ve
Neuroticism ➔ AP	0.470	5.317	0.000	Supported +ve
Neuroticism ➔ TEI	0.104	1.198	0.115	Not Supported

TEI, Trait Emotional Intelligence; AP, Academic Procrastination.

### Mediation analysis

To understand the relationship between personality traits and academic procrastination, present paper also explored mediating effect of trait emotional intelligence. To study total mediating influence by exploring total and indirect effects, researchers studied (Zhao et al., 2010) *t*-statistics, estimates, and 95% confidence interval. According to Zhao et al. (2010) and Hays and Scharkow (2013), if zero is not encompassed in bias corrected confidence interval mediation effect is considered substantial. For mediating hypotheses, the perusal of the Table 6 shows that the trait emotional intelligence partially mediates the relationship between openness and academic procrastination (*β* = 0.254, *t*-value = 8.769, *p* = 0.000), and between conscientiousness and academic procrastination (*β* = 0.199, *t*-value = 2.211, *p* = 0.024). In both cases, the mediating relationship is competitive. In case of agreeableness, the results reveals that trait emotional intelligence fully mediates the relationship between agreeableness and academic procrastination (*β* = 0.109, *t*-value = 1.998, *p* = 0.029). Further, the analysis shows that trait emotional intelligence does not mediates the relationship between extraversion and academic procrastination (*β* = 0.035, *t*-value = 1.201, *p* = 0.189), and neuroticism and academic procrastination (*β* = 0.042, *t*-value = 1.205, *p* = 0.114).

**Table 6:** Testing the pathways of mediation model.

Relationships	*β*	*T* statistics	*p* values	Result
Openness ➔ TEI ➔ AP	0.254	8.769	0.000	Partial mediation (competitive)
Conscientiousness ➔ TEI ➔ AP	0.199	2.211	0.024	Partial mediation (competitive)
Extraversion ➔ TEI ➔ AP	0.035	1.201	0.189	No mediation (direct effect)
Agreeableness ➔ TEI ➔ AP	0.109	1.998	0.029	Full-mediation (competitive)
Neuroticism ➔ TEI ➔ AP	0.042	1.205	0.114	No mediation (direct effect)

## Discussion

To the best of our knowledge, this is the first study conducted in Saudi Arabia to examine how trait emotional intelligence mediates the relationship between personality traits and academic procrastination among medical students. These findings corroborate and deepen prior insights, highlighting the significant role of both dispositional traits and emotional competencies in shaping academic behavior (Awad et al., 2023; Dong et al., 2022). The results demonstrated that conscientiousness, openness, and agreeableness were significantly negatively associated with academic procrastination, while extraversion and neuroticism showed positive associations. These findings are supported by previous findings who have established connection between academic procrastination and certain elements of the Big Five model particularly conscientiousness (Ljubin-Golub et al., 2019; Gao et al., 2021; Hidalgo-Fuentes et al., 2024) and neuroticism (Moslemi et al., 2020; Ocansey et al., 2022; Cao et al., 2025). Conscientiousness is characterized by a person’s ability to regulate their behavior through self-discipline, maintain order, work diligently, and follow established guidelines (Roberts et al., 2014). Our results revealed that conscientiousness had a notably strong negative association with academic procrastination and significantly predicted procrastinatory behavior in regression models. This outcome echoes findings from a number of recent investigations that emphasize a consistent negative link between conscientiousness and procrastination tendencies (Ljubin-Golub et al., 2019; Liu et al., 2020; Wang et al., 2021; Ocansey et al., 2022; Hidalgo-Fuentes et al., 2024). Highly conscientious individuals typically possess better impulse control and self-regulation (Wang et al., 2021), which serve as protective factors against procrastination (Yang et al., 2019; Cheng et al., 2023). Furthermore, their natural inclination toward being thorough, planning ahead, and managing time effectively contributes to their reduced likelihood of delaying academic tasks (Mirhashemi and Goodarzi, 2014). Interestingly, some personality theories have even classified procrastination as a component of low conscientiousness (MacCann et al., 2009).

Openness to experience, often linked to intellectual curiosity (McCrae, 1987) showed a significant negative correlation in our sample, consistent with the results of various prior studies (Day et al., 2003; Nadeem et al., 2016). This consistency suggested that students who score high in openness—marked by a strong desire to learn and intrinsic motivation (Ocansey et al., 2022)—tend to find studying enjoyable. As a result, they are generally less prone to procrastinate on academic assignments and homework (Steel and Klingsieck, 2016). Nonetheless, some research presents divergent findings, reporting either a positive association (Nadeem et al., 2016) or no clear relationship between openness and procrastination. Agreeableness was also significantly and negatively associated with academic procrastination. This negative association suggests that students with higher levels of agreeableness may be less likely to engage in academic procrastination, possibly to avoid letting down peers who depend on them in collaborative settings (Shaw and Choi, 2022; Guilera et al., 2018). Moreover, agreeableness may indirectly reduce procrastination through its influence on motivational regulation strategies. Individuals high in agreeableness often seek out and structure supportive learning environments, which may help minimize distractions and foster timely task completion, thereby decreasing the likelihood of academic procrastination (Ljubin-Golub et al., 2019).

Conversely, extraversion and neuroticism showed positive associations with academic procrastination. These findings are consistent with prior research (Freeman et al., 2011; Hen and Goroshit, 2014; Irfan et al., 2015; Wilt and Revelle, 2017; Abood et al., 2019; Bushra and Suneel, 2021; Zhang et al., 2022; Cao et al., 2025), which suggests that individuals high in extraversion may intentionally delay tasks to enhance excitement and stimulation. Rather than being disorganized, these individuals might seek the pressure of approaching deadlines as a source of motivation and arousal (Zhang et al., 2022; Freeman et al., 2011). Similarly, those with higher levels of neuroticism, characterized by anxiety, emotional instability, and self-doubt, may engage in procrastination as a coping mechanism to avoid task-related stress or fear of failure. In such cases, procrastination can serve as a temporary escape from academic demands, even though it ultimately increases anxiety and undermines performance. Together, these findings highlight that procrastination may serve different psychological functions depending on the underlying personality traits involved.

The most significant contribution of this study lies in exploring trait emotional intelligence as a mediator. In the present study, trait emotional intelligence is conceptualized as a trait-based construct, reflecting individuals’ self-perceived emotional abilities or emotional self-efficacy, in line with Petrides’ Trait emotional intelligence framework (Petrides, 2001; Petrides et al., 2007). Accordingly, trait emotional intelligence was assessed using a self-report measure, and the observed mediating effects should be interpreted as reflecting emotional self-perceptions and regulatory tendencies, rather than performance-based emotional abilities. Our results showed that trait emotional intelligence significantly mediated the effects of openness, conscientiousness, and agreeableness on academic procrastination. This indicates that students with these positive personality traits are more likely to possess emotional awareness and regulation, which buffers them from procrastination. These findings build upon earlier studies (Ferrando et al., 2011; Hjalmarsson and Dåderman, 2022; Awad et al., 2023), which suggest that higher emotional intelligence—conceptualized as emotional self-perceptions may reduce procrastination behaviors in students. Specifically, trait emotional intelligence partially mediated the effects of openness and conscientiousness, while fully mediating the link between agreeableness and procrastination. These results suggest that students with higher emotional self-efficacy may be better able to manage the emotional discomfort associated with academic tasks, consistent with emotion-regulation perspectives (Salovey and Mayer, 1990). Agreeable students, characterized by empathy and cooperation, may engage in fewer avoidance behaviors when supported by higher levels of trait emotional intelligence. The finding is reinforced by Parker et al. (2004), who found that students high in both agreeableness and EI are more likely to display academic resilience.

Conversely, trait emotional intelligence did not significantly mediate the impact of extraversion and neuroticism on procrastination. These traits may be linked to procrastination through mechanisms not captured by emotional intelligence—such as impulsivity, avoidance, or thrill-seeking behaviors (Rozgonjuk et al., 2023). This implies that while EI buffers certain maladaptive personality traits, it may be insufficient alone to counteract high emotional reactivity or impulsiveness. Importantly, the direct effect of trait emotional intelligence on procrastination was significant, indicating that independent of personality, students with higher trait emotional intelligence are less likely to procrastinate. This finding is in agreement with previous studies (Alghamdi et al., 2023; Balkis and Duru, 2009), which emphasizes EI as a cognitive-emotional resource that influences self-regulation and academic performance.

The current study is not without limitations. First, although a stratified approach was used to ensure balanced representation across academic years, participants were recruited on a voluntary basis from a single medical college in the Eastern Province of Saudi Arabia. This recruitment strategy may introduce selection bias and limits the generalizability of the findings to medical students in other regions or educational settings. Second, the study relied exclusively on self-report instruments, which are inherently dependent on participants’ self-awareness and may be affected by response and social desirability biases. Future research may reduce these limitations by incorporating objective measures or multi-method assessment approaches. Third, although socio-demographic characteristics were reported to describe the sample, they were not included as covariates nor formally examined using inferential statistical analyses; therefore, future studies may benefit from testing socio-demographic differences or incorporating these variables as covariates where theoretically and empirically justified. Lastly, measurement-related issues also pose a limitation. Although all instruments used in this study demonstrated acceptable to strong internal consistency (as indicated by Coefficient Alpha), some of the tools may require further validation to ensure their cultural relevance and suitability for use among Saudi medical students.

Despite its limitations, this study presents several meaningful implications for intervention and future research. The findings highlight valuable directions for educational psychology and student development programs. Notably, the observed partial and full mediation effects of trait emotional intelligence on the relationship between personality traits and academic procrastination suggest that interventions aimed at enhancing trait emotional intelligence —such as training in emotional regulation, self-awareness, and empathy—may be effective in reducing procrastination, particularly among students with high levels of neuroticism or low conscientiousness. Integrating such non-cognitive skill development into academic curricula, especially in high-stress disciplines like medicine, has the potential to bolster students’ academic resilience, emotional well-being, and overall performance.

Future research should consider employing longitudinal and experimental designs to establish causal relationships and assess the long-term efficacy of trait emotional intelligence-based interventions. Furthermore, extending this line of inquiry to diverse cultural and educational contexts could provide a broader understanding of how personality traits and trait emotional intelligence interact to influence academic behaviors globally. Finally, the development and integration of digital platforms or mobile-based trait emotional intelligence training modules could offer accessible, scalable, and cost-effective solutions for enhancing student support on a larger scale.

## Conclusion

This study adds to the expanding body of research investigating the psychological underpinnings of academic procrastination by emphasizing the influential role of personality traits and trait emotional intelligence. The findings reaffirm that individual differences in personality—particularly traits such as conscientiousness, openness to experience, and agreeableness—are significantly associated with students’ tendencies to procrastinate. Importantly, the study highlights trait emotional intelligence as a key mediating factor that helps explain how these personality traits influence procrastination behavior. Students who score high on trait emotional intelligence are better equipped to regulate their emotions, manage academic stress, and maintain motivation, thereby mitigating the negative effects of procrastination. This underscores the value of incorporating emotional skills training into medical education programs. By fostering emotional awareness, self-regulation, and interpersonal effectiveness, such interventions can empower students to adopt more adaptive study behaviors, enhance academic performance, and promote psychological well-being. Given the demanding nature of medical training, these insights offer practical implications for developing evidence-based strategies aimed at reducing procrastination and supporting students’ long-term academic and personal success.

## Data Availability

The raw data supporting the conclusions of this article will be made available by the authors, without undue reservation.
